# Machine Learning Enhances the Performance of Bioreceptor-Free Biosensors

**DOI:** 10.3390/s21165519

**Published:** 2021-08-17

**Authors:** Kenneth E. Schackart, Jeong-Yeol Yoon

**Affiliations:** 1 Department of Biosystems Engineering, The University of Arizona, Tucson, AZ 85721, USA; schackartk@email.arizona.edu; 2 Department of Biomedical Engineering, The University of Arizona, Tucson, AZ 85721, USA

**Keywords:** label-free biosensor, machine learning, support vector machine, artificial neural network, principal component analysis

## Abstract

Since their inception, biosensors have frequently employed simple regression models to calculate analyte composition based on the biosensor’s signal magnitude. Traditionally, bioreceptors provide excellent sensitivity and specificity to the biosensor. Increasingly, however, bioreceptor-free biosensors have been developed for a wide range of applications. Without a bioreceptor, maintaining strong specificity and a low limit of detection have become the major challenge. Machine learning (ML) has been introduced to improve the performance of these biosensors, effectively replacing the bioreceptor with modeling to gain specificity. Here, we present how ML has been used to enhance the performance of these bioreceptor-free biosensors. Particularly, we discuss how ML has been used for imaging, Enose and Etongue, and surface-enhanced Raman spectroscopy (SERS) biosensors. Notably, principal component analysis (PCA) combined with support vector machine (SVM) and various artificial neural network (ANN) algorithms have shown outstanding performance in a variety of tasks. We anticipate that ML will continue to improve the performance of bioreceptor-free biosensors, especially with the prospects of sharing trained models and cloud computing for mobile computation. To facilitate this, the biosensing community would benefit from increased contributions to open-access data repositories for biosensor data.

## 1. Introduction

The field of biosensing has exploded into nearly all areas of research, from medical applications [1] to environmental monitoring [2]. Some of the greatest appeals of biosensors are their specificity and sensitivity. These properties are primarily due to bioreceptors, which are selected for their inherent specificities such as enzymes [3], antibodies [4], and aptamers [5]. However, the very aspect that makes biosensors so specific and sensitive can also limit the sensor stability due to the degradation of the bioreceptor [6]. Additionally, as the bioreceptor is specific to an individual analyte, the particular sensor’s scope is limited to the specific analyte to which the bioreceptor can bind.

To obviate these issues, many nature-inspired sensors have emerged that are bioreceptor-free. Some of the most notable examples that have made great progress include the electronic nose (Enose) [7,8,9,10,11] and electronic tongue (Etongue) [12,13,14,15,16]. Additionally, surface enhanced Raman spectroscopy (SERS)-based sensors have demonstrated incredible chemosensing ability [17,18,19,20,21]. Without a bioreceptor, however, there is the risk of significantly compromised biosensor performance including the limit of detection (LOD) and specificity. Researchers have introduced machine learning (ML) to bioreceptor-free biosensors to bridge this trade-off gap, improving the LOD and specificity [22]. In a sense, ML can be used to take the place of a bioreceptor by reintroducing specificity during data analysis. This is made possible by powerful ML techniques capable of detecting subtle patterns in sensor responses.

While this approach has demonstrated success, there are still several challenges that these systems must overcome. A major challenge being faced is model generalizability. Since many models rely on subtle patterns in the data, they can be quite sensitive to underlying data changes. This can make the models susceptible to error when faced with sensor drift or replacing parts of the system [14].

Since the scope of this review is quite large and covers all bioreceptor-free biosensors that utilize ML, there are a few points to clarify. Many subsets of our scope have received thorough attention and review. For instance, the use of ML for Enose and Etongue [23,24,25,26,27] and SERS-based biosensors [28] have previously been described. Since the literature is rich in these areas, we realize that all recent original research cannot be adequately covered here. Rather, our intent is to provide a unified discussion of the relevant methods and challenges to give a bigger picture. We also would like to acknowledge that there is a complementary review in the literature discussing the use of ML in biosensing in general [29], but not for biosensors that are bioreceptor-free.

In this review, we will give the current state of using ML to enhance the performance of bioreceptor-free biosensors. Section 2 briefly introduces the types of biosensors that have most benefited from ML. Section 3 provides some background on machine learning algorithms and how their performance can be assessed. Section 4 covers electrochemical biosensors, with particular emphasis on Enose and Etongue. Successful methods are discussed as well as some of the challenges and how they are being addressed with ML. Section 5 discusses optical biosensors, notable for imaging- and SERS-based biosensors. Additional considerations and future perspectives are discussed in Section 6 including what currently prevents many of these systems from being commercialized and what directions may be taken. We also present some considerations on best practices for ML in biosensing, especially regarding communication of methods and reproducibility.

## 2. How Biosensors Can Benefit from Machine Learning

Biosensors in the classic definition are sensors that utilize a bioreceptor such as antibody, enzyme, peptide, nucleic acid, etc. A bioreceptor binds to a target biological molecule and generates a signal when coupled with a transducer. Biosensors have evolved to a wide range of transducer types including electrochemical, optical, and spectroscopic biosensors. Traditionally, it is the bioreceptor that provides specificity and sensitivity to the biosensor. Increasingly, however, researchers are developing biosensors that lack a specific bioreceptor. A typical example is a semi-specific chemical sensor array, termed Enose (from gas), or Etongue (in solution). Since such a sensor’s specificity is not provided by the bioreceptor, a fingerprinting technique is used to recognize signal patterns indicative of a particular analyte. Frequently, machine learning techniques are employed to detect these patterns and provide specificity.

The use of machine learning to enhance the performance (e.g., specificity, sensitivity, and LOD) of bioreceptor-free biosensors is not limited to chemical sensor arrays. It has been employed in various biosensor mechanisms. Some of the most famous examples aside from Enose and Etongue are imaging-based biosensors and SERS-based biosensors. Additionally, the use of machine learning for biosensors is not limited to those that lack bioreceptors. Cui et al. [29] cover several examples of traditional biosensors employing machine learning to enhance performance.

Table 1 provides an overview of the tasks for which machine learning has been applied, the specific algorithms used, and the relevant papers. More information on the algorithms themselves can be found in Section 3. Additionally, Table 2 gives a comparison of each of the major biosensing mechanisms including data type and appropriate feature engineering and ML methods. All information in Table 2 comes from Table 1 and serves as a higher-level summary.

## 3. A Brief Tour of Machine Learning

In simple terms, machine learning aims to learn patterns in data to make predictions on new data. Generally, this prediction is either categorical classification (into one of a set of classes) or regression (continuous numerical output). In machine learning terms, the data used for prediction (i.e., biosensor data) are termed features or predictors. The set of features associated with one “observation” (e.g., biosensor data from one sample) is termed the feature vector.

### 3.1. Feature Engineering

Frequently, the predictor variables (feature vector) are not the raw biosensor data. One of the most challenging parts of using machine learning is the construction of the feature vector from the raw data. This process is termed feature engineering and mostly entails finding the relevant information from the data to aid the machine learning algorithm’s performance. Common feature engineering steps include denoising, normalization, and rescaling.

One of the most powerful feature engineering processes is dimension reduction. This reduces a large number of features to a smaller number of features while minimizing information loss. Perhaps the most common method of dimension reduction is principal component analysis (PCA) [74], which reduces the original set of variables to a smaller set of independent variables termed principal components (PCs). The effectiveness of PCA to represent the data can be assessed by the amount of variance in the data explained by the PCs. Since PCA determines the PCs based on the eigenvectors’ directions in the feature space, data must first be centered and rescaled to avoid bias toward those variables with a larger magnitude. Another common dimension reduction algorithm is linear discriminant analysis (LDA), which also produces a smaller number of variables but is supervised and optimally maximizes class separation [75]. Other more complex dimension reduction methods exist including artificial neural networks (ANN), as discussed in Section 3.3. ANN is typically used as a supervised machine learning method, while it has occasionally also been used for dimension reduction.

### 3.2. Unsupervised vs. Supervised

The two broad categories of machine learning algorithms are unsupervised and supervised [76]. In unsupervised methods, data labels are not provided during model training, while in supervised methods, they are. An example of an unsupervised algorithm is cluster analysis, used to group similar data. Unsupervised methods are less common in biosensing since we generally know what kind of prediction(s) we would like the model to make. A notable exception is PCA, as mentioned in Section 3.1. While PCA may be considered an unsupervised machine learning method, its use has recently been limited to dimension reduction (one of feature engineering processes) prior to supervised machine learning analyses.

### 3.3. Classification Algorithms

Among the supervised methods, classification algorithms are some of the most well-known. Classification gives prediction in the form of a class label (e.g., which bacteria species is present), thus, the output is inherently categorical. Briefly, some of the most common classification algorithms are presented in the following.

**Figure 1:**
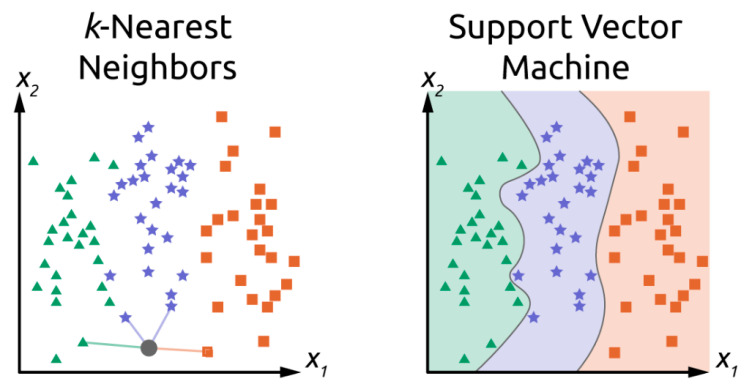
Comparison of classification technique using *k*-NN and SVM. In *k*-NN, four nearest neighbors are shown contributing to the gray point’s assignment. Classification of the gray point is the blue star class. In hypothetical SVM with nonlinear kernel, new data are classified in which region the point lies. In both examples, the feature space consists of two dimensions. Classification could be, for example, bacterial species like *E. coli*, *Salmonella* spp., *Pseudomonas* spp., *Staphylococcus* spp., *Enterococcus* spp., etc. In practical applications, the feature space has many more dimensions, where decision boundaries for SVM are hyperplanes in the (*n*−1) dimension for an *n*-dimensional feature vector.

*k-nearest neighbors* (*k-NN*): One of the simplest classification algorithms, *k*-NN is a distance-based classifier. Class is predicted as the most common class of the *k*-nearest neighbors in the feature space [77]. In the example shown in Figure 1, the feature space is two dimensional (with variables *x*_1_ and *x*_2_) and the value of *k* is 4. In *k*-NN, the number of neighbors used for assignment, *k*, is a hyperparameter (i.e., a model parameter that is not optimized during the training process itself). As with most ML models, hyperparameter selection may strongly influence performance [78].

*Support vector machine* (*SVM*) is a non-probabilistic, binary, linear classifier [79]. SVM relies on the construction of hyper-plane boundaries in the feature space to separate data of different classes. Although SVM itself only accounts for linear separation of classes (i.e., hyper-plane boundaries must be “flat”), the data may be mapped to a higher-dimensional feature-space using the “kernel trick” [80]. Some of the most common kernels are radial basis function and Gaussian. When the hyperplane boundaries are projected back into the original feature space, they allow for non-linear boundaries, as shown in Figure 1. Additionally, there are methods allowing SVM to be used for multi-class prediction [81]. The placement of hyperplanes is determined by minimizing the distance between the hyperplane and several of the points closest to the boundary between classes. SVM’s robustness against outliers is improved by a soft margin. This allows for a certain quantity of misclassifications, which are presumably outliers, to improve the separation of the other observations [82]. While SVM shows resilience against outliers and performs well in high-dimension feature spaces, it is prone to over-fitting, especially when using non-linear kernels [83]. Overfitting is when the model performs well on training data but performs poorly when generalized to unseen data.

*Linear discriminant analysis* (*LDA*): In addition to dimension reduction, LDA can be used for classification. Other related algorithms allow for non-linear classification such as quadratic discriminant analysis (QDA) [84]. One of the limitations of LDA and its relatives is that they assume the data are normally distributed.

**Figure 2:**
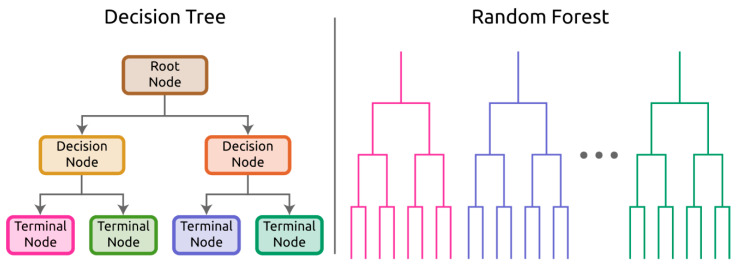
Decision tree (DT) showing nodes at which binary decisions are made on features. Terminal node dictates model prediction. Actual DTs have many more nodes than shown here. Random forest (RF) shown as a series of distinct decision trees.

*Decision tree* (*DT*) and *random forest* (*RF*): In tree-based models such as decision tree (DT), the feature vector starts at the tree’s “trunk,” and at each branching point a decision is made based on the learned decision rules. The end classification would then be at the terminal or “leaf” node that the instance results. DTs can be used for classification and regression [85]. When the target variable is categorical, it is referred to as a classification tree; when the target variable is numerical and continuous, it is referred to as a regression tree [86]. Random forest (RF) is so called because it can be considered a forest of decision trees (Figure 2) [87]. There are many RF architectures, but in all instances, the classification from each decision tree contributes to the overall classification for an observation.

**Figure 3:**
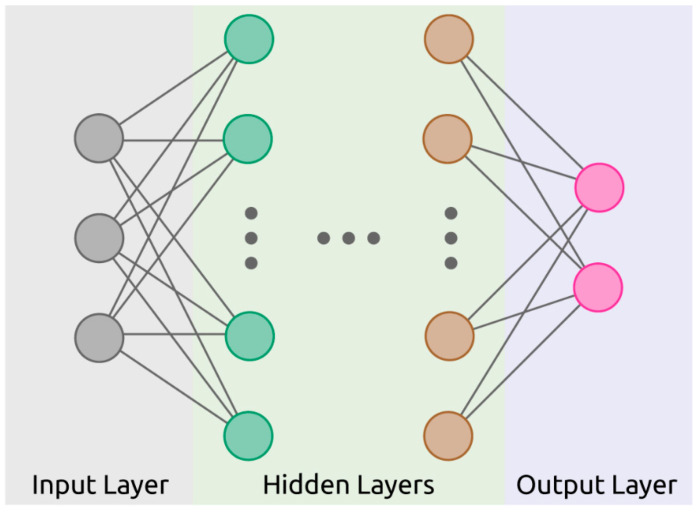
Artificial neural network (ANN) showing nodes of the input, hidden, and output layers.

*Artificial neural network* (*ANN*) draws inspiration from biological neural networks (i.e., neurons in the brain) and is composed of a collection of connected nodes called artificial neurons (see Figure 3). ANNs can be used for classification and regression. As mentioned earlier, ANN can be used for dimension reduction prior to supervised machine learning. There are a large variety of ANN structures such as (1) recurrent neural network (RNN) [88], (2) extreme learning machine (ELM) [89], and (3) deep learning algorithms such as the convolutional neural network (CNN) [90], deep belief network [91], and back-propagation neural network (BPNN) [92]. “Deep” indicates several hidden layers. ANN architectures have many hyperparameters such as the number of hidden layers, connectedness, and activation functions [93].

One of the aspects that makes ANN so powerful is that features do not need to be well-defined real numbers. This allows them to excel at working with data such as images for which extracting numerical features would be difficult and inefficient. One limitation of ANNs is that they require a large amount of data for effective training. In some settings, training data sparsity can be mitigated through a generative adversarial network (GAN) using back propagation [94].

Common classification model performance metrics are accuracy, precision, sensitivity (also known as recall), specificity, and *F*1. For binary classification with labels “positive” and “negative”, they are defined as follows:

(1) $$accuracy=\frac{TP+TN}{TP+TN+FP+FN}\;$$

(2) $$precision=\frac{TP}{TP+FP}\;\;$$

(3) $$sensitivity=\frac{TP}{TP+FN}\;\;\;\;$$

(4) $$specificity=\frac{TN}{TN+FP}\;$$

(5) $$F1=\frac{2\times precision\times sensitivity}{precision+sensitivity}$$

where *TP* is true positive, *TN* is true negative, *FP* is false positive, and *FN* is false negative.

### 3.4. Regression Algorithms

In contrast to classification, the prediction made by a regression algorithm is a numeric value from a continuous scale (e.g., glucose concentration in blood). A simple regression example fits a linear model of the form *y* = *mx* + *b*, where a model is built for the prediction of the output variable *y* based on the input variable *x*, and the coefficients *m* and *b* are “learned” from the data. The learning is typically done by the least-squares regression approach, minimizing the sum of the squared residuals. The following are some of the most common regression algorithms.

*Multilinear regression* (*MLR*) is a simple regression model, which expands the above linear model example, accounting for multiple input variables. This model shows how it can be difficult to determine when an algorithm becomes sophisticated enough to be considered “machine learning”.

*Support vector regression* (*SVR*) is an adaptation of SVM used for regression problems. Like SVM, SVR can utilize kernels to allow for non-linear regression. An advantage of SVR over traditional regression is that one need not assume a model that might not be accurate. For instance, with linear regression, there is an assumption that the data distribution is linear. SVR does not require such pre-determined assumptions [95].

*Regression tree* is an adaptation of DT for regression. Regression tree has the advantage that it is non-parametric, implying that no assumptions are made about the underlying distribution of values of the predictors [86].

*Artificial neural network* (*ANN*) is also widely used for regression problems, and many varieties exist, some of which were mentioned previously.

A large variety of metrics exist for regression model performance. Since there are too many to define here, for further reading, we suggest the study by Hoffman et al. [96] to learn more. Some of the most common metrics are briefly presented here. Root mean squared error (RMSE) and mean absolute error (MAE) have the benefit that their units are the same as the output (predicted) variables, but this makes the metrics less universally understandable. Normalized root mean squared error (NRMSE) partially resolves that. Coefficient of determination, *R*^2^, on the other hand, is unitless and *R*^2^ ≤ 1, where a value near 1 is generally considered good performance (although this is a bit oversimplified).

### 3.5. Model Performance Assessment

Frequently, researchers will try various models and compare their performance. The value of the performance metrics listed above can be treated as random variables and statistical analyses can be used to test hypotheses regarding which model is better [96]. While this sounds simple, it can be nuanced: for instance, when working with a classification model, which metric is most important for your application? In some cases, specificity may be more important than accuracy, for instance. Additionally, when using statistical tests to compare model performances, certain assumptions are made, and their validity should be assessed such as when using NRMSE, as it is assumed that noise affecting the output is random and normally distributed.

The best practice for model selection, tuning, and performance assessment is to split the data into 3 sets: training, testing, and validation. For example, if the database consists of 1000 observations, 100 (10%) are assigned to the validation set and the remaining 900 (90%) are split between the training and test sets as 810 (90%) for training, 90 (10%) for testing. The model is then trained on the labeled training set. Model selection and hyperparameter tuning is conducted based on model performance when challenged using the test set. In addition to train–test splitting, cross-fold validation can be used on the training set when tuning hyperparameters or comparing models [97]. Train–test splitting and cross-validation are most important when you intend to generalize the model to predict new, unseen data [96]. Final model performance validation is conducted on the validation set, which should not be used until all model selection and hyperparameter tuning have been completed.

## 4. Electrochemical Bioreceptor-Free Biosensors

Since their inception, electrochemical biosensors have become extremely popular. In traditional electrochemical biosensors, the bioreceptor interacts with the target to generate a signal at the electrical interface. A widespread scheme is an enzyme (e.g., glucose dehydrogenase or glucose oxidase) interacting directly with the target analyte (e.g., glucose), catalyzing a redox reaction that generates a signal at the electrical interface [98]. Electrical interfaces include metal electrodes, nanoparticles, nanowires, and field-effect transistors (FET) [99]. 

It is also possible to eliminate the biorecognition element (=bioreceptor, e.g., an enzyme) in electrochemical biosensors. Voltametric sensors described in Section 4.1 can detect biomolecules based on direct interaction with the electrical interface [30]. Electrical impedance spectroscopic biosensors can also detect subtle differences in a solution or material’s electrical impedance, as discussed in Section 4.2. Alternatively, we can use an array of chemical or physical sensors varying the electrical interface to create multi-dimensional data. Machine learning-based pattern recognition is used to identify the target analyte. Two of the most common sensor arrays are termed Enose and Etongue, which are covered in Section 4.3.

### 4.1. Cyclic Voltammetry (CV)

**Figure 4:**
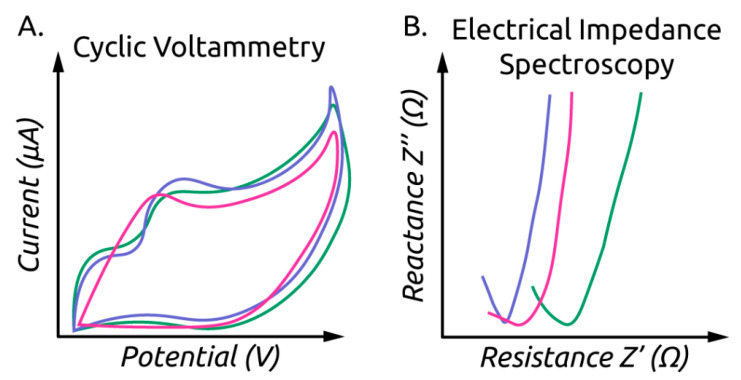
(**A**) Hypothetical cyclic voltammograms for three samples. (**B**) Hypothetical Nyquist plot obtained through EIS showing curves for three samples.

Voltammetry sensors apply electric potential to a “working” electrode and measure the current response, which is affected by analyte oxidation or reduction [100]. Cyclic voltammetry (CV) is a specific voltammetry technique in which the potential is swept across a range of values, and current response is recorded. These CV curves (cyclic voltammograms) can serve as a fingerprint of the sensor response. A typical CV curve is shown in Figure 4A.

CV biosensors often employ bioreceptors to provide specificity in the interaction between target analyte and electrode surface. However, there has also been research on utilizing more complex electrode surface structures and modifications to allow for semi-specific interaction with the target analyte without the need of a bioreceptor. Sheng et al. [30] describe a compound electrode utilizing Cu/PEDOT-4-COOH particles for CV detection of the phytoinhibitor maleic hydrazide. They found that several regression models had poor performance for modeling the sensor current response with respect to target concentration. However, they employed an ANN with great success for the same regression task. The result is that their detection range is broader than comparable methods by an order of magnitude at each extreme (detection range = 0.06–1000 μM and LOD = 0.01 μM).

### 4.2. Electrical Impedance Spectroscopy (EIS)

**Figure 5:**
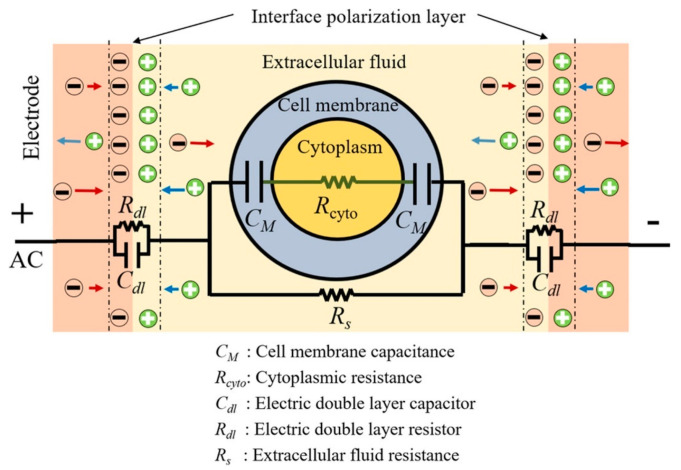
Equivalent circuit diagram of single cell suspension. Reproduced with permission from [102] without modification. Copyright 2020 John Wiley and Sons.

Electrical or electrochemical impedance spectroscopy (EIS) is an analytical technique that provides a fingerprint of the electrical properties of a material. EIS is performed by applying a sinusoidal electric potential to a test sample and recording the impedance (both resistance and reactance expressed in a complex number) over a range of frequencies [101]. Frequently, an equivalent circuit model is fitted to EIS data to provide a fingerprint of the material properties [101]. Figure 5 shows an equivalent circuit diagram for EIS being performed on a single cell suspension. An example EIS spectrum is shown in Figure 4B. It is the classification and regression on such fingerprints that machine learning tends to be well suited.

A simple example of this is the use of *k*-NN on EIS data for the detection of adulteration in milk [41]. In this work, the feature space was composed of resistance at a certain temperature and pH. They demonstrated good accuracy of 94.9%. However, the data were highly imbalanced, and in the example classification plot [41], one of the three unadulterated samples were misclassified, a 66% specificity.

More robust classification has been performed using SVM. One example is for the assessment of avocado ripeness [43]. This work describes using PCA for feature extraction, resulting in two PCs that explain >99.3% of the variance. SVM is then used for classification based on the first two PCs. SVM for EIS was also described by Murphy et al. [44] for classification of malignant and benign prostatic tissue. However, instead of using PCA for feature extraction, equivalent electrical circuit model parameters were used as predictors. The feature vector size was 2160, consisting of four electrical features for each of eleven frequencies across multiple electrode configurations. Classification was also performed on electrical impedance tomography (EIT) data from the same samples using SVM. Both showed good classification performance, though the authors mention that EIT may be preferable since the measurements are not dependent on probe electrical properties, and thus can be compared more easily to other studies.

While SVM is renowned for its tolerance of outliers, this is a trade-off in that data points not near the boundary between classes do not contribute to defining class attributes. However, ANNs preserve more of this information for prediction. When the number of observations or predictors are small, this can lead to overfitting. However, with sufficient data size, ANNs can preserve predictive information and be robust against outliers and overfitting. These attributes have been utilized for EIS based classification of breast tissue [40,42]. Both works use the same publicly available dataset of EIS measurements from freshly excised breast tissue [103], made available on the University of California, Irvine (UCI) Machine Learning Repository [104]. The dataset contains nine spectral features from EIS. Daliri [40] describes using three ELMs, each with different numbers of nodes, and feeding the output of the three ELMs (extreme learning machines) into SVM for classification. This method showed improved performance over previous methods for the same dataset such as LDA [105]. Helwan et al. [42] compared both BPNN and radial basis function network (RBFN) for the same task. Both methods showed an improvement over ELM-SVM as described by Daliri [40], with RBFN performing better than the BPNN including improved generalizability (i.e., classification performance on new data).

It is seen that in the case of EIS classification, node-based models have shown improved performance over other models. This can be seen most clearly when comparing classification accuracy for those methods that utilized the same dataset. The RBFN and BPNN had the highest classification accuracy, with 93.39% and 94.33%, respectively [42]. The next best performance was achieved by the ELM-SVM, achieving 88.95% accuracy [40]. These results show marked performance increase over LDA [105]. Model performance is greatest in those models that do not utilize distance for classification (i.e., SVM and LDA). While distance-based classifiers are robust to outliers, in these EIS datasets, performance benefitted by node-based classification.

### 4.3. Enose and Etongue

**Figure 6:**
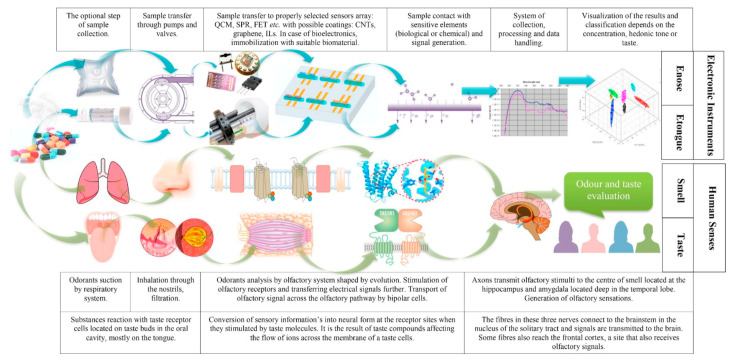
Comparison of operation principle of Enose and Etongue, and the analogy to the biological systems. Reproduced with permission from [27] without modification. Copyright 2019 Elsevier.

Enose and Etongue are named in analogy to their respective animal organs. Both sensor types rely on an array of semi-specific sensors, each of which interacts to a different degree with a wide range of analytes. Figure 6 shows a comparison between Enose and Etongue alongside the analogy to their respective biological systems [27,106]. The sensor arrays can be composed of any variety of sensors. The following chemical gas sensors have been used in Enose systems: metal oxide (MOX) gas sensor, surface or bulk acoustic wave (SAW and BAW) sensors, piezoelectric sensor, metal oxide semiconductor field-effect transistor (MOSFET) sensor, and conducting polymer (CP) based sensor [107]. Similarly, a variety of sensors can be employed in Etongue systems such as ion-selective field-effect transistor (ISFET) and light-addressable potentiometric sensor (LAPS) [108].

Analyte presence, or a more general attribute such as odor or taste, is detected through pattern recognition of the sensor array response. For pattern recognition on this naturally high-dimensional data, machine learning techniques are an obvious choice. Scott et al. provided a relevant and succinct paper on data analysis for Enose systems [23]. As discussed in Section 3 of this review, feature engineering is critical in any machine learning pipeline. Yan et al. [24] provide a review article on the feature extraction methods for Enose data. For non-linear feature extraction of Etongue data, Leon-Medina et al. [46] give a great comparison of seven manifold learning methods.

A vast number of papers exist detailing such systems and their use of machine learning. As such, it would be infeasible to cover all of them adequately. For this review, a higher-level analysis is presented by looking at the conclusions reached in the review papers covering this topic as well as a few notable examples of specific papers. Of particular interest is which algorithms had the most success with Enose and Etongue sensors or applications.

A common task of Enose is the prediction of “scent”, which is a classification problem. Before the application of the classification algorithm, it is common to perform dimension reduction. PCA is the most common choice for this task, although independent component analysis (ICA, a generalization of PCA) has shown success [25]. PCA has been shown to improve the performance over classification algorithms alone for the piezoelectric Enose [25]. The two classifiers most commonly in use are SVM [109,110] and various ANN methods [25,111]. In addition to classification problems, Enose may be used for analyte concentration prediction. One example is the use of MOS (metal oxide semiconductor) gas sensors for formaldehyde concentration assessment. In this case, the back-propagated neural network (BPNN) outperformed radial basis function network (RBFN) and SVR [33]. In another instance, with the single nickel oxide (NiO) gas sensor, PCA with SVR was utilized for harmful gas classification and quantification [32]. In cases where the amount of data are not large, SVM may be advantageous over node-based models (ANNs) for its resilience against outliers and overfitting.

While Enose and Etongue systems have shown great promise for non-destructive analytical devices, there are challenges that have limited their use in commercial settings. Several challenges involve changes in the sensor data, which affect the performance of the trained model. A common phenomenon is when the sensor array response changes over time or upon prolonged expose under identical conditions. Such change in sensor response is referred to as sensor drift and can greatly affect the trained models’ performance [14]. Another way in which the sensor response may change is if a sensor in the array becomes defective and must be replaced, as it is difficult to replace it with one that responds identically, largely due to variability in manufacturing [112,113]. For both challenges, time consuming and computationally expensive recalibration may be necessary.

The issue of needing retraining due to underlying data distribution changes is commonly addressed through transfer learning in many machine learning settings. Transfer learning is a computational method for minimizing the need for retraining when either the data distributions change (e.g., sensor array response to an analyte) or the task changes (e.g., new classes of analytes are being detected).

**Figure 7:**
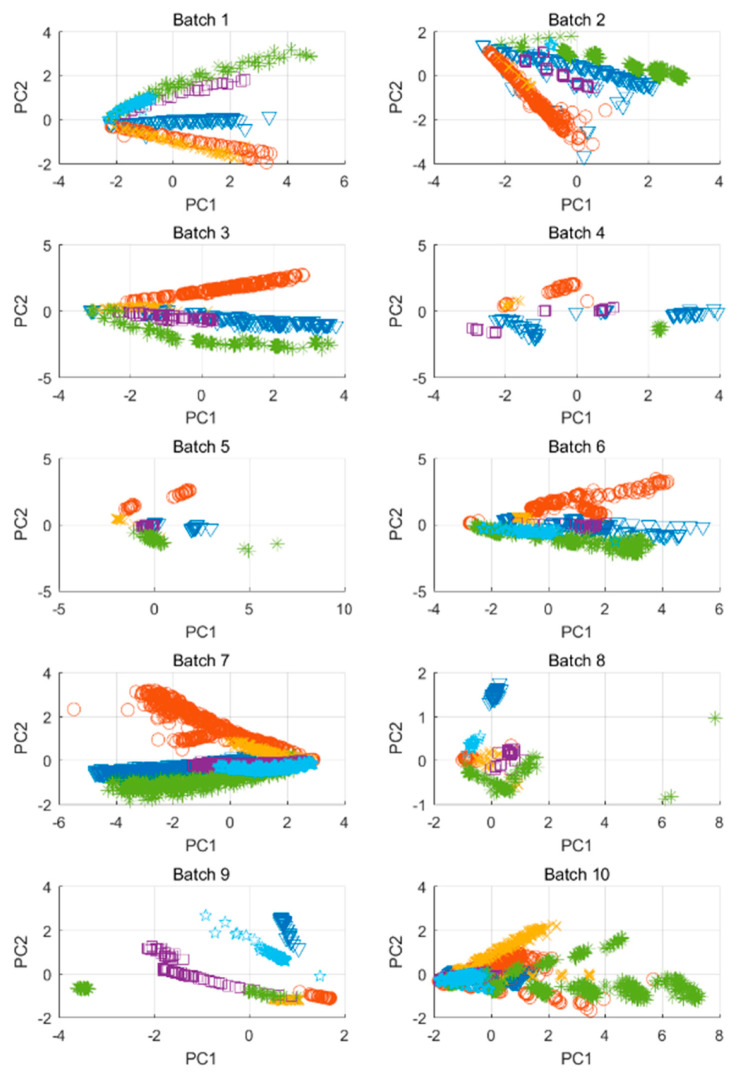
Gas sensor drift dataset from [36]. Each color represents a different gas. Each panel represents a measurement “batch” at various times spanning 36 months. Reproduced from [36] without modification, under Creative Commons Attribution 4.0 License.

Transfer learning has been extensively employed to counter Enose sensor drift and reduce the need for complete retraining [35,36,37,38]. It has also been used to reduce the deleterious effect of background interference [39,114]. Although several of the above papers [35,36,38,39] demonstrate the efficacy of their approach on a shared sensor drift dataset shown in Figure 7 [115], ranking of the methods is difficult due to inconsistent benchmarking metrics. As mentioned previously, the data distribution may also change due to replacing a sensor with a new sensor, or when attempting to apply a trained model to a theoretically identical array with differences due to manufacturing variability. Transfer learning, specifically using ANN, has demonstrated decent recalibration [116].

One instance of utilizing transfer learning for target task change was demonstrated by Yang et al. by training an Enose classifier on wines (source task) and applying it to classify Chinese liquors (target task) while only retraining the output layer [34]. Interestingly, transfer learning has been used much less commonly for Etongue systems, although they also face sensor drift. However, Yang et al. utilized transfer learning to improve the generalizability of their Etongue [45]. In this work, they demonstrate the superiority of their transfer learning trained CNN over other methods such as BPNN, ELM, and SVM for tea age classification.

**Figure 8:**
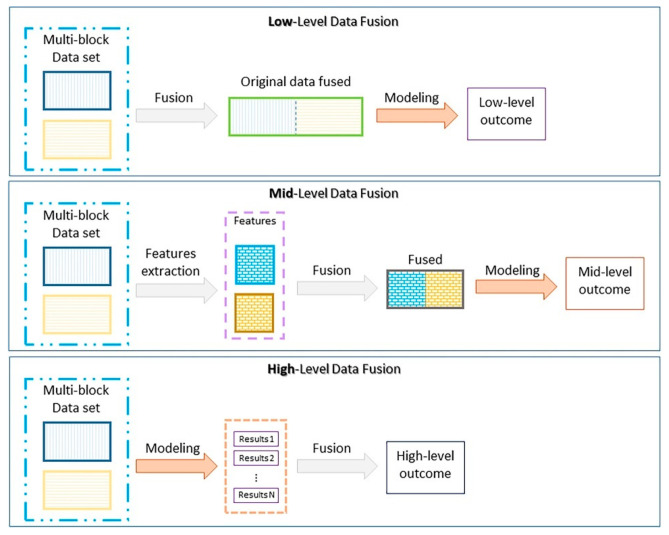
General scheme depicting the main differences among low-, mid-, and high-level data fusion. Reproduced with permission from [118] without modification. Copyright 2019 Elsevier.

A trend that has been gaining traction is data fusion to combine Enose and Etongue systems. The value of this can again be appreciated in how closely the senses of smell and taste are linked in animals [117], complementing each other to provide the most accurate assessment. Similarly, by using information from both Enose and Etongue, better analysis can be conducted. As illustrated in Figure 8, data fusion can be performed at three levels: low, mid, and high [118]. Recently, mid-level fusion schemes have shown promising results for fusion of Enose and Etongue data [119,120], especially when performing PCA on the two systems and using those features for fusion before model training [121,122,123]. Such systems have also benefitted from the inclusion of a computer vision system in data fusion [121,124].

Currently, another class of systems exist with the same goals as Enose and Etongue that utilize biochemical recognition elements, termed bioelectronic nose (bEnose) and tongue (bEtongue). These devices utilize biological elements such as taste receptors, cells, or even tissues for sensing [106,125]. These systems show impressive selectivity and sensitivity, especially when coupled with nanomaterials to aid in signal transduction from the biochemical recognition element [106,126]. Their major challenges, as with most biosensors, is stability and reproducibility of the biological element [106]. For these reasons, Enose and Etongue remain popular for their sensor stability. Continued efforts are necessary to improve sensitivity closer to their bioelectronic counterparts, especially regarding sensor design and feature extraction methods.

With such a large variety of sensors in use for Enose and Etongue systems, data processing can vary significantly. Of particular interest is finding appropriate feature extraction methods [23,24]. A huge variety of machine learning classification and regression methods have been employed, both on unsupervised dimensionally reduced feature vectors and classically extracted features. Transfer learning methods have been successful in allowing target task change with minimal retraining, especially when using node-based models. However, the challenges posed by sensor drift and manufacturing variability are still significant and will likely remain a focus for researchers over the next several years.

### 4.4. Summary of Electrochemical Bioreceptor-Free Biosensing

Many electrochemical bioreceptor-free biosensors employ chemical or physical sensor arrays coupled with machine learning. These are most obvious in Enose and Etongue systems, inspired by nature (humans and animals). Other systems generate multivariate spectral data also coupled with machine learning. In both cases, machine learning models can aid in analyte classification or quantification. Especially when using distance-based models, choice of feature extraction method is important to optimally capture the features relevant to the task (i.e., classification or regression). Node-based models, primarily ANNs often require less feature extraction pre-processing as this step is built into the model learning. Additionally, node-based models offer a great solution to target task change and noise elimination through transfer learning, often aided by integration through the back-propagation step so that only the final layer needs to be refined [34].

## 5. Optical Bioreceptor-Free Biosensors

The mechanisms of optical detection in biosensing are diverse. A classic example is the colorimetric lateral flow assay [127,128,129]. Mechanisms beyond colorimetry include fluorescence [130,131,132], luminescence [133], surface plasmon resonance [134], and light scattering [135,136].

Machine learning has been widely employed in optical biosensors. An example with similarities to Enose and Etongue is the bacterial bioreporter panel. Each bacterial bioreporter responds to target analytes in a semi-specific manner. Machine learning is used to discover patterns in the bioreporter panel response and relate them to analyte presence or concentration [137,138]. However, this review’s focus is to discuss cases in which the bioreceptor is absent, so such sensors are not covered in detail.

Another prevalent use of machine learning for analyzing images as biosensor data is for image processing, especially segmentation [139,140,141,142]. The literature is rich in reviews on machine learning for image segmentation, and this technology is in no way specific to biosensors, so this review will not discuss those examples. However, the topic is essential to many biosensors, so it must be mentioned.

### 5.1. Imaging

Imaging sensors utilize an array of optical sensors such as a CMOS array (complementary metal-oxide-semiconductor array; the most used image sensor for digital cameras). Images of the specimen can be used to identify the target presence and concentration as the molecules exhibit different coloration, fluorescence, or light scattering, with varying morphology and spatial distribution. In this manner, several imaging biosensors have been developed to eliminate the need for labels and bioreceptors.

**Figure 9:**
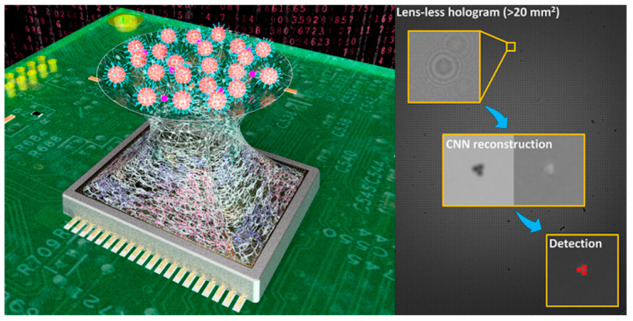
Lensless hologram reconstruction via CNN for particle aggregation detection. Reprinted with permission from [55] without modification. Copyright 2019 American Chemical Society.

A growing field of imaging-based biosensors utilizes lens-free imaging techniques [143,144]. Since the images from lens-free imaging are not in focus, computational techniques are needed for image reconstruction, the most common of which is deep learning (mostly based on ANN with “deeper” layers) [53,54,145]. Lens-free imaging may be used to detect the aggregation of particles caused by bioreceptor–analyte interaction [55] (Figure 9). However, an exciting application is the direct, label-free classification of particles by lensless holography. Wu et al. [56] presented a lensless holography biosensor for classifying pollen and spores. As with many of these systems, a CNN was used for image reconstruction. In this work, another CNN was used to classify the particles, yielding > 94% accuracy.

Another work on the imaging classification of pollen utilizes multispectral imaging [58]. Again, a CNN was trained for classification, and a species-averaged accuracy of 96% was achieved for 35 plant species.

**Figure 10:**
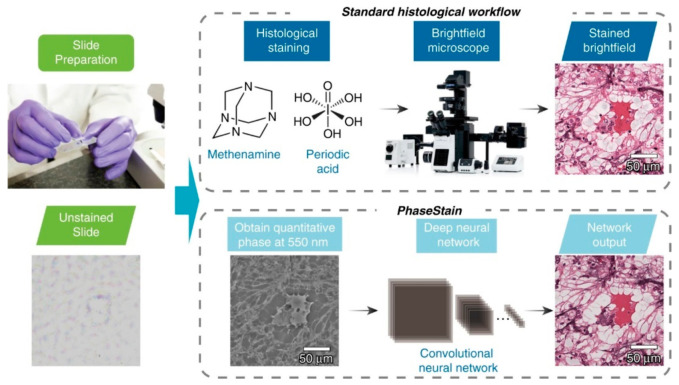
A quantitative phase image of a label-free specimen is virtually stained by a deep neural network, bypassing the standard histological staining procedure that is used as part of clinical pathology. Reproduced from [59] without modification under Creative Commons Attribution 4.0 License.

Artificial neural networks (ANNs) have also found great success in the developing field of digital staining. Hematoxylin and eosin (H&E) stain is the most common stain for histology [146]. However, the quality of tissue staining is subject to many factors that can affect the diagnosis. Digital staining is an alternative in which tissue sections are imaged unstained, and a trained model generates an image simulating stained tissue (Figure 10). Deep learning has been applied for digital staining on images acquired from a variety of methods including quantitative phase imaging [59], Fourier transformed infrared spectroscopy (FTIR) [52], and multi-modal multi-photon microscopy [57]. To overcome the issue of data scarcity and overfitting, researchers have frequently employed generative adversarial neural network (GAN) for medical imaging [147], which has shown promising results for digital staining model training [148]. Additionally, transfer learning has improved the model’s generalizability to multiple domains [50].

Fluorescence-based imaging biosensors are also worthy of mention. Sagar et al. [51] presented a microglia classification based on fluorescence lifetime utilizing ANN.

The applications of imaging biosensors are extensive. Indeed, the scope is too large to analyze all papers in this review. However, of particular importance to imaging biosensors is the ANN, especially the CNN. This preference is expected since CNN has shown exceedingly good performance in a variety of image classification contexts [149,150].

### 5.2. Colorimetry

One class of optical biosensors is the colorimetric biosensor. Currently, the applications of machine learning to enhance the performance of bioreceptor-free colorimetric biosensors are limited. This limitation is because the colorimetric biosensors (most notably lateral flow assays) mostly utilize bioreceptors (e.g., antibodies, enzymes, and aptamers) [98]. One example of such a bioreceptor-free biosensor is non-invasive plant disease diagnosis by Li et al. [49]. They utilized an array of plasmonic nanocolorants and chemo-responsive organic dyes that interact with volatile compounds from the plant. Their technique is similar to Enose and Etongue since it is a fingerprinting approach to the array response for classification. They used PCA, but do not cite an actual classifier, although they give performance metrics such as accuracy. At this time, it is unclear how the classification was performed on the PCA-transformed data.

Most colorimetric biosensors do not require machine learning due to their simplicity for readout. However, the arrays of bioreceptor-free (semi-specific) colorimetric sensors require machine learning-based classification in a way similar to Enose and Etongue. In these instances, they will likely benefit from the same treatment, namely dimension reduction by PCA and SVM classification.

### 5.3. Spectroscopy

**Figure 11:**
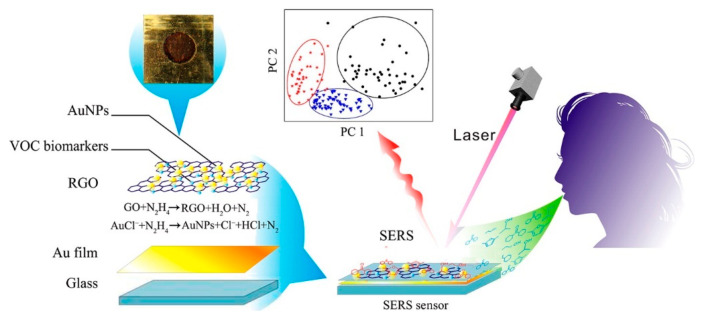
SERS sensor for analysis of breath VOC biomarkers utilizing AuNPs. Reprinted with permission from [154] without modification. Copyright 2016 American Chemical Society.

Of the spectroscopic biosensing techniques, surface-enhanced Raman spectroscopy (SERS) has shown great success [151,152]. SERS is a vibrational surface sensing technique that enhances Raman scattering based on surface characteristics. Briefly, SERS utilizes incident laser light to induce inelastic scattering (Raman scattering) from the target analyte. The intensity of the Raman scattering is enhanced by interaction with the conduction electrons of metal nanostructures (SERS substrate). The enhancement of the Raman scattering is what makes SERS so sensitive. Researchers have reported enhancement factors of up to ten or eleven orders of magnitude [153]. Figure 11 illustrates a SERS sensor for the analysis of breath volatile organic compound (VOC) biomarkers [154]. Due to the complex nature of the obtained spectral signal, various machine learning algorithms have been used to process SERS data in multiple contexts [28].

Although bioreceptors may be used to allow for specific binding of the target analyte to the SERS sensing surface [155,156], direct detection is also possible. Robust classification and regression algorithms can bring specificity and sensitivity to these biosensors. A simple yet effective method for SERS based quantification is partial least squares regression (PLSR). PLSR has been used for a variety of quantification applications such as biofilm formation monitoring [69], blood serum methotrexate concentration [63], aquaculture toxins [62], and food antiseptics [66]. PLSR has the advantage of model simplicity with well-defined parameters, but it may be insufficient in modeling data with significant sources of noise. 

**Figure 12:**
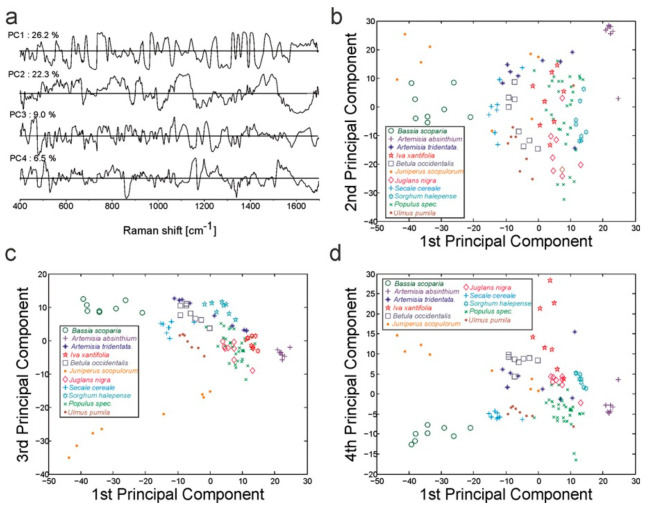
PCA results using the spectral range of 400–1700 cm^−1^ of 112 average SERS spectra from 14 different commercially available pollen species. Loadings of the first four PCs (**a**) as well as the scores of the first and second (**b**), first and third (**c**), and first and fourth PC (**d**) are shown. PCA was done with standardized first derivatives of the mean spectra of 500 vector-normalized spectra. Reprinted with permission from [72] without modification. Copyright 2016 John Wiley and Sons.

Since the spectra have high dimensionality, dimension reduction is a frequent preprocessing step (Figure 12). PCA is again popularly used as a dimension reduction or feature extraction step [60,61,64,65,68,70,71,73], or for exploratory analysis [62,72,157]. Once the spectra are remapped using PCA, a classifier or regression model is employed such as an extreme learning machine (ELM) [71], LDA [68], SVM [60,64,73], PLSR [65], or ANN [70]. An alternative to dimension reduction is utilizing the high dimensionality spectral data directly with a node-based algorithm such as ANN [72,158,159] and CNN [160,161].

The reusability and generalizability of the trained models are often limited. Spectral response is affected not just by analyte presence but surface structure. Therefore, for the model to be reused on a new SERS biosensing dataset, the surface characteristics must be very similar. In terms of transfer learning, this is an issue of changes in the underlying data distributions. However, if the surface structure methods are well documented and reproducible, transfer learning could be employed on a spectral library [28]. Ideally, researchers could contribute to this library in an open-access manner and use these spectra for model training. In this case, the quality of the attached metadata would be a crucial factor.

Clearly, machine learning has been used extensively in the context of SERS sensors. The most common pipeline is to perform unsupervised dimensionality reduction/feature extraction for which PCA is generally the preferred method. Less consistency is seen in the algorithms used for classification and regression. Alternatively, ANNs can be used directly on the data, and the advantage of one approach over the other is not clearly illustrated in the literature. We anticipate, however, that like in the case of electrochemical sensors, node-based models would allow for more efficient transfer learning to accommodate target task change.

### 5.4. Summary of Optical Bioreceptor-Free Biosensing

A variety of optical sensing methods have benefited from machine learning techniques, with the preferred method being dependent on the data type. For image type data, CNN is the most obvious choice for its ability to detect features as well as reconstruct images obtained by lensless systems. For spectral data, the approach is similar to spectral data obtained with electrochemical sensors. In those instances, dimensionality reduction coupled with a classification/regression algorithm may perform nearly as well as node-based methods. Indeed, they may be preferable in instances where the quantity of training data is small.

## 6. Considerations and Future Perspectives

Biosensor research has shown great success and promise. For both systems with and without bioreceptor, ML has demonstrated huge success in going from large, complex sensor datasets to getting meaningful measurements and classification of analytes. However, in many of these systems, a key challenge is consistency in device manufacturing. This manifests itself regarding sensor reproducibility for Enose and Etongue, or as substrate reproducibility for SERS. Since the models used to process these data often rely on subtle signals in the data, even small changes in sensor response characteristics can lead to poor performance. These issues have effectively limited widespread commercial adoption of these technologies. There has been some success in accommodating these inconsistencies through computational methods, notably with transfer learning for Enose. More work, both from a manufacturing and computational standpoint, needs to be done before many of these systems are robust enough for widespread adoption.

One area in which these systems have pushed to increase commercial potential is through miniaturization and modularity. There have been efforts with several of the methods presented here to develop compact standalone devices that rival their bulkier counterparts in terms of performance [16,47,162,163,164,165,166]. We believe that cloud computing may be a key element to the success of these endeavors. Some of the models in use, especially for image-based sensors, are computationally expensive. By offloading the computational work to cloud computing, the device footprint imposed by processing and memory needs is greatly reduced.

A central question is what the relative advantages and disadvantages are between systems that utilize a bioreceptor and those that do not. A key advantage of those that eliminate the bioreceptor addresses one of the barriers to commercialization—manufacture variability. By eliminating the bioreceptor, device manufacture is simplified, and may decrease manufacture variability. Additionally, sensor longevity is generally improved because the long-term stability of the bioreceptor is often limited [6]. However, to match LOD and specificity of bioreceptors, improvements must be made. Nanomaterials show promise for improving device performance [167].

There have been studies that attempt to gain the advantages of both systems by creating artificial bioreceptors, notably nanomaterials with enzymatic properties referred to as nanozymes [168,169]. While exciting progress has been made in this field, current nanozyme-based biosensors have inferior catalytic activity and specificity to their biological alternatives [170,171]. Nanozyme catalytic activity is also currently limited to oxidase-like activity [171]. If researchers can broaden nanozyme activity and improve selectivity, these biosensors may become a competitive alternative for biological bioreceptors.

In addition to device considerations, there are computational challenges to consider. Although some ML algorithms have been in use for decades such as PCA and SVM, the field of ML is advancing rapidly with new algorithms being described frequently. While many areas are quick to adopt the new methods, improper usage is common and certainly not limited to biosensing. Some common mistakes are inappropriate data splitting, hidden variables serving as bad predictors, and mistaking the objective of the model [172]. Great emphasis must be placed on the importance of reporting appropriate performance metrics. A great example of a misleading metric is reporting accuracy on highly imbalanced data such as in Durante et al. [41]. It can often be difficult to determine if the proper pre-processing and model assumption checks are being performed. This may be centering and re-scaling prior to PCA, or normality checks for LDA.

Some of these issues can be solved with better methods reporting, especially regarding computational methods. Certain key details are frequently left out, making critical evaluation difficult and reproducibility impossible [173]. One of the most striking examples from the literature described herein is reporting classification metrics, without reporting what classifier was used on PCA processed data [49]. Perhaps the best way to make methods clear and reproducible is to release all associated code, preferably publicly.

Increased availability in general can greatly improve this field. More open access repositories of training sets may allow researchers to improve model robustness by exposing them to more diverse datasets [16]. Some examples currently exist such as the gas sensor drift dataset [115] and the EIS breast tissue dataset [103], both available in the UCI Machine Learning repository [104]. One vision would be to have large repositories of gas sensor responses to many analytes under various experimental conditions. Models could be trained on such repositories to improve generalizability. Ideally, with such repositories and improved manufacturing consistency, trained models could be shared directly and need only minimal recalibration.

## 7. Conclusions

In this review we have explored the ways in which bioreceptor-free biosensors can benefit from ML methods. Robust ML models bring specificity and accuracy to array-based biosensors such as Enose and Etongue by learning the patterns in the sensor responses. Notably, PCA has shown great performance as a feature extraction technique for these systems. Similar power of PCA has been demonstrated for optical biosensors that generate spectra such as Raman spectra or SERS. ANNs using deep learning generate impressive results for imaging-based sensors including lensless holography and digital staining. ML has also been used in creative ways such as for data fusion of multiple biosensors, and transfer learning for noise correction, sensor drift compensation, and domain adaptation.

However, many practical challenges still exist. Many of the methods presented here are not widely used in commercial settings. This is due to many reasons including variability in manufacturing and the ability to make compact versions of the biosensors while maintaining performance. ML models that can adapt to differences in sensor response are at an advantage, and transfer learning shows promise to be part of the solution.

In recent years, ML has garnered strong research interest in many fields including biosensing, as evidenced in this review. If this review has inspired interest to learn more about how machine learning is being used for one of the methods presented here, we encourage you to seek more specific reviews for the subject. There are great reviews in the literature, many of which were referenced, that take a closer look at the methods presented in this review.

## Figures and Tables

**Table 1:** Machine learning tasks and algorithms used in biosensing.

Biosensing Mechanism	Task	Target	Algorithm	Ref.
ELECTROCHEMICAL				
CV	Regression	Maleic hydrazide	ANN	[30]
CV	Classification	Industrial chemicals	LSTM, CNN	[31]
Enose	Feature extraction	Harmful gases	PCA	[32]
	Classification		DT, RF, SVM	
	Regression		SVR	
Enose	Regression	Formaldehyde	BPNN	[33]
Enose	Classification	Chinese wines	BPNN	[34]
	Target task change	Chinese liquors	Transfer learning	
Enose	Sensor drift compensation for classification	Gases	JDA	[35]
			DTBLS	[36]
			TrLightGBM	[37]
			ELM	[38]
Enose	Sensor drift compensation & noise reduction	Bacteria	ELM	[39]
EIS	Classification	Breast tissue	ELM + SVM	[40]
EIS	Classification	Milk adulteration	*k*-NN	[41]
EIS	Classification	Breast tissue	RBFN	[42]
EIS	Feature extraction	Avocado ripeness	PCA	[43]
	Classification		SVM	
EIS & EIT	Classification	Prostatic tissue	SVM	[44]
Etongue	Taste classification	Tea storage time	CNN	[45]
	Increase generalizability		Transfer learning	
Etongue	Feature Extraction	Beverages	*t*-SNE	[46]
	Classification		*k*-NN	
Etongue	Classification	Cava wine age	LDA	[47]
Etongue	Regression	Black tea theaflavin	Si-CARS-PLS	[48]
OPTICAL				
Colorimetric	Classification	Plant disease VOCs (blight)	PCA	[49]
Diff. contrast microscopy	Digital staining & domain adaptation	Leukocytes	GAN	[50]
Fluorescence imaging	Classification	Microglia	ANN	[51]
FTIR imaging	Digital staining	H&E stain	Deep CNN	[52]
Lens-free imaging	Image reconstruction	Blood & tissue	CNN	[53,54]
		Herpes		[55]
Lens-free imaging	Image reconstruction & classification	Bioaerosol	CNN	[56]
Multi-modal multi-photon microscopy	Digital staining & modal mapping	Liver tissue	DNN	[57]
Multispectral imaging	Classification	Pollen species	CNN	[58]
Quantitative phase imaging	Digital staining	Skin, kidney & liver tissue	GAN	[59]
Raman spectroscopy	Feature extraction	Thyroid dysfunction biomarker	PCA	[60]
	Classification		SVM	
TLC-SERS	Feature extraction	Histamine	PCA	[61]
	Quantification		SVR	
SERS	Exploratory analysis	Malachite green & crystal violet	PCA	[37,62]
	Quantification		PLSR	
SERS	Quantification	Methotrexate	PLSR	[63]
SERS	Classification	Oil vs lysate spectra Leukemia cell lysate	*k*-means clustering	[64]
	Dimension reduction		PCA	
	Classification		SVM	
SERS	Dimension reduction	Levofloxacin	PCA	[38,65]
	Regression		PLSR	
SERS	Quantification	Potassium sorbate & sodium benzoate	PLSR	[66]
SERS	Dimension reduction & regression	Congo red	PCR	[39,67]
SERS	Dimension reduction	Mycobacteria	PCA	[40,68]
	Classification		LDA	
SERS	Quantification	Biofilm formation	PLSR	[41,69]
SERS	Feature extraction	Non-structural protein 1	PCA	[70,71]
	Classification		BPNN, ELM	
SERS	Exploratory analysis	Pollen species	PCA, HCA	[72]
	Classification		ANN	
SERS	Feature extraction	Human serum	KPCA	[73]
	Classification		SVM	

Note. CV = cyclic voltammetry; ANN = artificial neural network; LSTM = Long short-term memory; PCA = principal component analysis; DT = decision tree; RF = random forest; SVM = support vector machine; SVR = support vector regression; BPNN = back-propagation neural network; JDA = joint distribution adaptation; DTBLS = domain transfer broad learning system; GBM = gradient boost machine; ELM = extreme learning machine; EIS = electrical impedance spectroscopy; EIT = electrical impedance tomography; *k*-NN = k-nearest neighbor; RBFN = radial basis function network; CNN = convolutional neural network; *t*-SNE = *t*-distributed stochastic neighbor embedding; Si-CARS-PLS = synergy interval partial least square with competitive adaptive reweighted sampling; FTIR = Fourier transform infrared; VOC = volatile organic compound; GAN = generative adversarial network; DNN = deep neural network; TLC = thin layer chromatography; SERS = surface enhance Raman spectroscopy; PLSR = partial least squares regression; PCR = principal component regression; LDA = linear discriminant analysis; HCA = hierarchical cluster analysis; KPCA = kernel principal component analysis.

**Table 2:** Summary of data types and useful ML methods for biosensing mechanisms.

Biosensing Mechanism	Description of Data	Feature Extraction	ML Model
CV	Cyclic voltammogram		ANN, LSTM, CNN
EIS	Nyquist plot	PCA	*k*-NN, ELM, SVM, RBFN
Enose	Multivariate	PCA	DT, RF, ELM, SVM, BPNN
Etongue	Multivariate	PCA, *t*-SNE	LDA, *k*-NN, CNN, PLS
Lens-free imaging	Image		CNN
Digital staining	Image		Deep learning, GAN
SERS	Spectrum	PCA, KPCA	PLSR, LDA, SVM, SVR, BPNN, ELM

## Data Availability

This study did not report any data.
